# Insights into Resistance Mechanisms of Inhibitors to Mps1 C604Y Mutation via a Comprehensive Molecular Modeling Study

**DOI:** 10.3390/molecules23061488

**Published:** 2018-06-20

**Authors:** Yuan Chen, Wenquan Yu, Cui-cui Jiang, Jin-gui Zheng

**Affiliations:** 1 College of Crop Science, Fujian Agriculture and Forestry University, Fuzhou 350003, China; katecy@163.com; 2 Institute of Agricultural Engineering and Technology, Fujian Academy of Agricultural Sciences, Fuzhou 350003, China; ywq333@163.net (W.Y.); yumin793@163.com (C.-c.J.)

**Keywords:** Mps1, C604Y, molecular modeling, resistance mechanisms

## Abstract

Mono-polar spindle 1 (Mps1/TTK) represents a protein kinase reported to be vital for cell division processes and is generally regarded as an attractive target for the treatment of hepatocellular carcinoma, breast carcinoma, and colon cancer. However, the C604Y mutation has been linked to acquired resistance. Recently, three potential small-molecule inhibitors of Mps1 (i.e., reversine, NMS-P715, and its derivative Cpd-5) were reported for the C604Y mutation that exhibit significant resistance to NMS-P715 and Cpd-5, but retain affinity for reversine. In this study, classical molecular dynamic (MD) simulations, accelerated MD (aMD) simulations, and umbrella sampling (US) simulations were performed to illustrate the resistance mechanisms of inhibitors to Mps1. The classical MD simulations combined with free energy calculations revealed that reversine features similar binding affinity characteristics to both Mps1^WT^ and Mps1^C604Y^, but both NMS-P715 and Cpd-5 feature much higher binding affinities to Mps1^WT^ than to Mps1^C604Y^. The major variations were shown to be controlled by electrostatic energy and the conformational change of A-loop-induced entropy increased. The large conformational changes of Mps1^C604Y^ bound to NMS-P715 and Cpd-5 were also observed in aMD simulations. The US simulation results further suggest that reversine and Cpd-5 both exhibit similar dissociation processes from both Mps1^WT^ and Mps1^C604Y^, but Cpd-5 and NMS-P715 were found to dissociate more easily from Mps1^C604Y^ than from Mps1^WT^, thus a reduced residence time was responsible for the inhibitors resistance to the C604Y mutation. The physical principles provided by the present study may provide important clues for the discovery and rational design of novel inhibitors to combat the C604Y mutation of Mps1.

## 1. Introduction

In eukaryotic cells, the process of cell mitosis has to be carefully choreographed in a detailed fashion to ensure the correct separation of genetic material to the two daughter cells. In this process, the spindle assembly checkpoint (SAC) represents an evolutionarily highly conserved mitotic control mechanism that is specifically required for proper segregation of chromosomes [1]. An SAC guarantees that a cell is not in a state of division until all sister chromatids align to the metaphase plate and are exactly captured by the mitotic spindle, resulting in the bipolar amphitelic attachment of chromosomes [2]. One of the main components of SAC is the mono-polar spindle 1 (Mps1, also known as TTK), a dual-specificity serine/threonine kinase that plays a leading role in the transition from metaphase to anaphase [3]. Due to the vital role of Mps1 in cellular function, an imbalance of Mps1 activity is often detrimental to cell survival. For instance, when Mps1 is depleted, mitosis proceeds regardless of proper chromosomal alignment, leading to aberrations in chromosome counts [4]. It has been well documented that a number of tumor types express abnormally high levels of Mps1. Overexpression of Mps1 in cancer cells has been suggested to prevent further gains or losses of chromosomes during progressive cell division. Therefore, it is not surprising that Mps1 is upregulated in a wide range of human tumor types, and that higher levels correlate to higher histological grade, invasion, and poorer patient survival in hepatocellular carcinoma, breast cancer, glioblastoma, and colon cancer, as well as pancreatic ductal adenocarcinoma [5,6,7].

**Figure 1:**
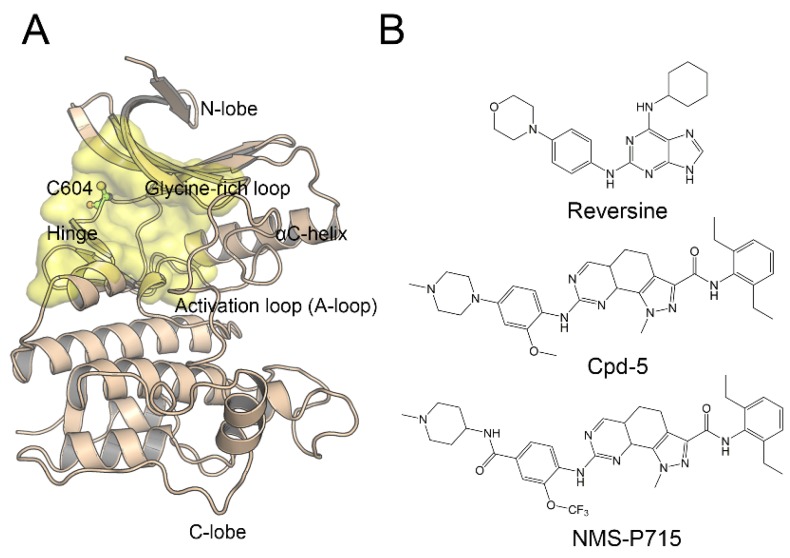
Structure of Mps1 and representative inhibitors. (**A**) Overview of Mps1 structure (PDB code: 5NTT), C604Y mutation is colored green ball-and-stick model. The binding pocket is the colored yellow surface; (**B**) Chemical structures of reversine, Cpd-5, and NMS-P715.

Subsequent studies have prompted significant interest in the development of Mps1 inhibitors as an anticancer treatment that targets the conserved adenosine triphosphate (ATP) binding site. To date, numerous compounds have been developed and patented. Various inhibitors have been shown to exhibit anti-proliferative activity in tumor cells [8,9,10,11]. The kinase domain of Mps1 adopts a two lobe architecture, including a smaller N-terminal lobe and a larger C-terminal lobe connected by a flexible kinase hinge, and the C604Y mutation is located in the kinase hinge (Figure 1A). The N-terminal lobe consists of six-stranded β-sheets, a crucial regulatory αC-helix and a phosphate bind loop (P-loop). The C-terminal lobe features a standard structure, including a β sheet of two strands adjacent to the N-terminal lobe, seven α-helices, a catalytic loop, and an activation loop (i.e., A-loop) encompassing residues 676–685 which are usually difficult to be resolved. In addition, phosphorylation of conserved A-loop is vital for kinase activity [12]. The ATP binding pocket is located in the cleft between the N-terminal and C-terminal lobes (Figure 1A, yellow surface) [13]. However, similar to other kinases, point mutations, which mostly occur in the conserved ATP binding site of Mps1 (Figure 1A), dramatically attenuate the therapeutic efficiency of Mps1 inhibitors. Previous studies have shown that relapsed tumors often express the C604Y, C604F, C604W, I153M, and S611R point mutation [11,14,15].

Recently, Hiruma et al. [14] reported Mps1^C604Y^ and Mps1^C604W^ raised resistance to two closely related compounds, NMS-P715 and its derivative Cpd-5; however, not to the well-studied Mps1 inhibitor, reversine (Figure 1B). Among them, the inhibitors of Cpd-5 and NMS-P715 inhibit the C604Y mutation with a worse IC_50_ or K_D_ value than C604W. Moreover, the C604Y mutation confers resistance more moderately to Cpd-5 than to NMS-P715, but reversine exhibits equivalent activities against wild-type Mps1 (Mps1^WT^) as well as the C604Y (Mps1^C604Y^) mutation. NMS-P715 has been characterized to suppress the growth of medulloblastoma cells, a malignant brain tumor commonly occurring in children. The anti-proliferative activity of NMS-P715 has also been demonstrated in breast, renal, and colon cancer cell lines [16,17]. Cpd-5, a derivative of NMS-P715, has been shown to exhibit higher potency and better tolerance towards wild type (WT)/C604Y than NMS-P715 [14,18]. Reversine was first reported to promote the stem cell de-differentiation. Past reports have shown that the main target of reversine is Aurora B kinase and the A_3_ adenosine receptor [19,20]. Recently, it has been reported that reversine does not only act on Aurora B, but also Mps1. In reality, the principle target of reversine is Mps1 and the potency of reversine on Mps1 was shown to be 10–20 fold higher than that on Aurora B [21,22].

Based on the crystal structures of the Mps1^C604Y^/reversine, Mps1^C604Y^/Cpd-5, and Mps1^C604Y^/NMS-P715 complexes, it is generally thought that the C604Y mutation is located at the kinase hinge and may block the key protein-ligand interactions [14]. Nevertheless, this explanation seems to be relatively ambiguous. A dynamic molecular understanding of resistance mechanism is of great importance to overcome the problem of resistance to Mps1 inhibitors, and may provide vital clues for inhibitor development for the treatment of various solid cancers. Therefore, in this study, classical molecular dynamic (MD) simulations, accelerated molecular dynamic (aMD) simulations, and enhanced sampling simulations (umbrella sampling, US) were carried out to shed light on the inhibitor resistance mechanism caused by the C604Y mutation via three reported inhibitors (reversine, Cpd-5, and NMS-P715). Classical MD simulations combined with dynamical cross-correlation (DCC) analysis, and binding free energy calculations were utilized to help recognize the impact of the C604Y mutation on the flexibility and dynamics of basic parts of Mps1 to inhibitor binding. Furthermore, we quantitatively highlighted the key protein-ligand interactions related to inhibitor resistance. Meanwhile, aMD simulations in conjunction with free energy map calculations were applied to provide the possibility of studying the conformational space in more detail in order to explore the local energy minima that is hidden in classical MD simulations. In addition, US simulations were performed as proof of principle of the dissociation processes of reversine, Cpd-5, and NMS-P715 from the WT and C604Y mutated Mps1. Potentially, the comprehensive analysis results may guide and provide important clues for the design of novel Mps1 inhibitors with improved characteristics to combat inhibitor resistance.

## 2. Results and Discussion

### 2.1. Structural Properties Studied by Classical MD Simulations

**Figure 2:**
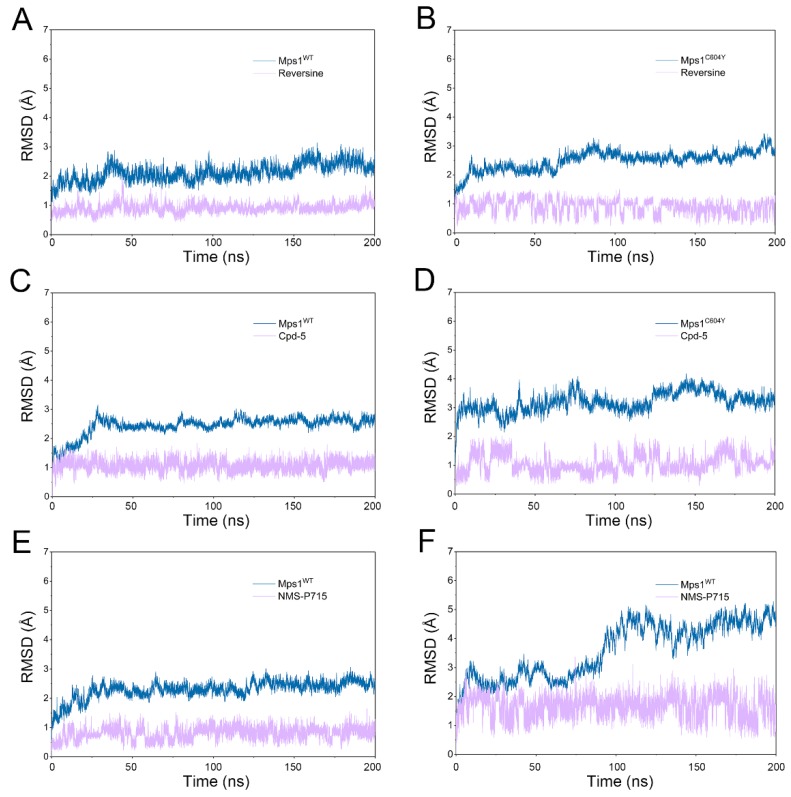
Toot-mean-square deviation (RMSD) analysis of reversine, Cpd-5 and NMS-P715 in Mps1^WT^ and Mps1^C604Y^ from classical molecular dynamic (MD) simulations. (**A**) Mps1^WT^/reversine; (**B**) Mps1^C604Y^/reversine; (**C**) Mps1^WT^/Cpd-5; (**D**) Mps1^C604Y^/Cpd-5; (**E**) Mps1^WT^/NMS-P715; (**F**) Mps1^C604Y^/NMS-P715.

To monitor the convergence of classical MD simulations, the root-mean-square deviation (RMSD) values of C_α_ for six systems during production using classical MD simulations were plotted as a function of time (Figure 2). As illustrated in Figure 2A,B, the RMSD curves for the proteins of Mps1^WT^ and Mps1^C604Y^ bound to reversine exhibited excellent stability characteristics with small fluctuations after simulation for ~ 50–100 ns. The curves for reversine in both Mps1^WT^ and Mps1^C604Y^ oscillated with minute fluctuations (~0.9 Å), indicating that reversine constrained the structural flexibility of both Mps1^WT^ and Mps1^C604Y^. The RMSD curves for Mps1^WT^ bound to Cpd-5/NMS-P715 also exhibited excellent stability after simulation for ~20–50 ns, with average RMSD values of 2.7 Å and 2.6 Å, respectively. Moreover, both Cpd-5 and NMS-P715 proved to be highly significantly stable during the entire simulations (Figure 2C,E). On the contrary, those for the C604Y mutation systems were found to be different compared to the WT systems. As shown in Figure 2D,F, the average RMSD value for both proteins and ligands of Mps1^C604Y^/Cpd-5 and Mps1^C604Y^/NMS-P715 were more fluctuant than others systems, implying the unstable nature of Mps1^C604Y^ bound to Cpd-5/NMS-P715.

**Figure 3:**
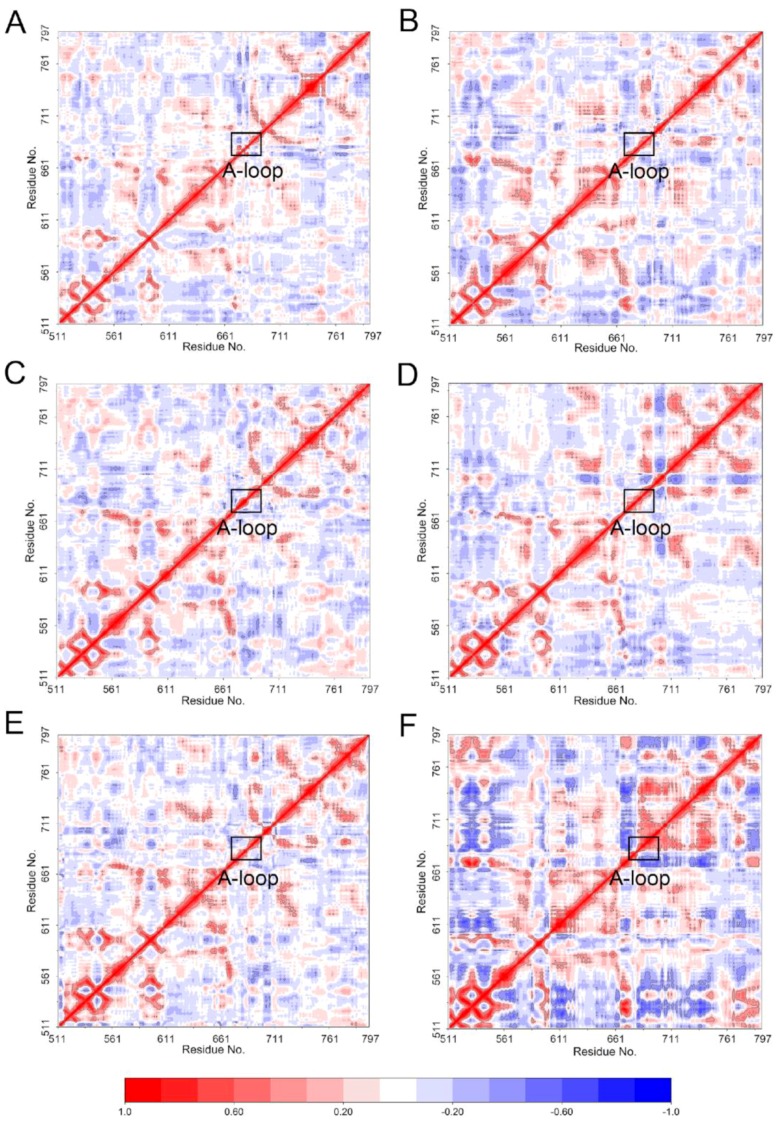
Dynamical cross-correlation (DCC) analysis of fluctuations of residues from classical MD simulations. (**A**) Mps1^WT^/reversine; (**B**) Mps1^C604Y^/reversine; (**C**) Mps1^WT^/Cpd-5; (**D**) Mps1^C604Y^/Cpd-5; (**E**) Mps1^WT^/NMS-P715; (**F**) Mps1^C604Y^/NMS-P715.

In order to assess the dynamic correlation differences for different simulated systems, the movement of all pairs of C_α_ atoms for all the simulated systems was performed by dynamical cross-correlation (DCC) analysis. This way, the pairwise correlations in the motions of the residues averaged all the sampled conformations during MD simulations were calculated. The positive regions (colored in red, ranging from 0 to −1) represented correlation motions between residues, and the negative regions (colored in blue, ranging from 0 to 1) referred to anti-correlation motions of specific residues. As shown in Figure 3, except for the diagonal regions which describe the correlation between residues and their own, the correlative motions in most regions of Mps1^WT^ and Mps1^C604Y^ shared high similarities with few highly correlated motions when bound to reversine (Figure 3A,B). However, there were significant differences between Mps1^WT^ and Mps1^C604Y^ when bound to Cpd-5/NMS-P715 (Figure 3C–F). In the C604Y mutation systems, the correlated and anti-correlated motions were found to be stronger than in the WT systems. Particularly, the A-loop regions of Mps1^C604Y^ bound to Cpd-5/NMS-P715 were highly correlated and anti-correlated motions that exhibited amplified fluctuations when compared with Mps1^WT^. In addition, the correlated and anti-correlated motions of Mps1^C604Y^/NMS-P715 were stronger than those for Mps1^C604Y^/Cpd-5, suggesting the dynamic and unstable A-loop may be responsible for the different degree of resistance to Cpd-5 and NMS-P715.

**Figure 4:**
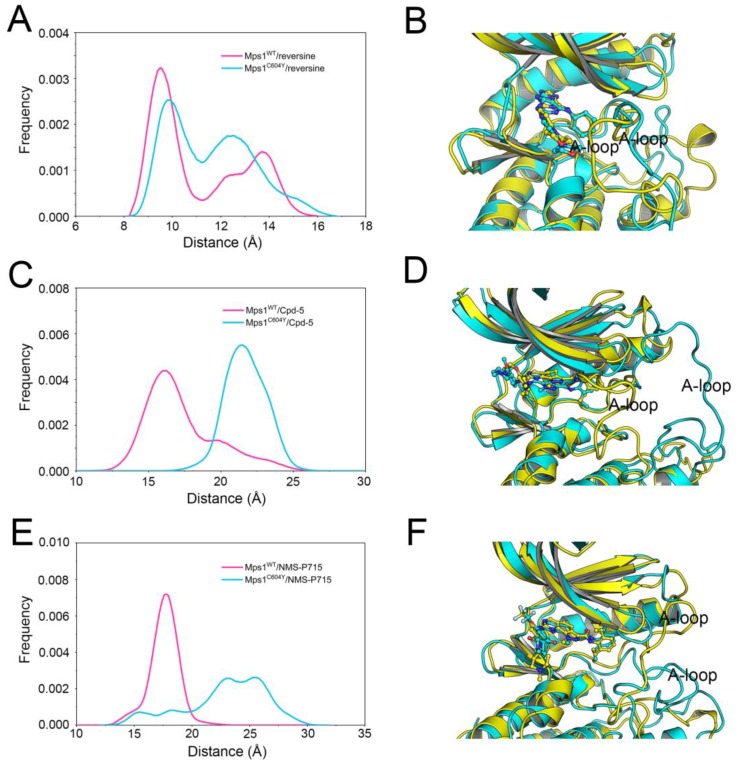
The distribution of the opening degree of the A-loop and alignment of the most populated between Mps1^WT^ and Mps1^C604Y^. The opening degree was defined as the center of mass of the A-loop to the center of mass of the inhibitor. The distribution of the opening degree of the A-loop between (**A**) Mps1^WT^/reversine and Mps1^C604Y^/reversine, (**C**) Mps1^WT^/Cpd-5 and Mps1^C604Y^/Cpd-5, (**E**) Mps1^WT^/NMS-P715 and Mps1^C604Y^/NMS-P715. Alignment of the most populated conformations between (**B**) Mps1^WT^/reversine and Mps1^C604Y^/reversine, (**D**) Mps1^WT^/Cpd-5 and Mps1^C604Y^/Cpd-5, (**F**) Mps1^WT^/NMS-P715 and Mps1^C604Y^/NMS-P715.

Next, the distribution of the opening degree of the A-loop, which represents the degree of the opening of the A-loop, was studied. The distribution of the opening degree of the A-loop rather than one representative snapshot or last snapshot from classical MD simulations was applied due to the flexibility of the A-loop, and the modelled initial conformations may not be in a proper minimum. The degree of opening of the A-loop was defined as the center of mass of the A-loop to the center of mass of the inhibitor. As illustrated in Figure 4A, the A-loop region of Mps1^WT^ and Mps1^C604Y^ bound to reversine showed a very similar distribution of the opening degree. The alignment of the most populated conformations between Mps1^WT^/reversine (yellow) and Mps1^C604Y^/reversine (cyan) showed a highly similar pattern with minor adjustments of the A-loop (Figure 4B). For comparison, those for Cpd-5/NMS-P715 exhibited a significant difference where the C604Y mutation systems showed a higher distribution of opening degree than the WT systems (Figure 4C,E). Furthermore, it was found that the conformational changes of the A-loop of the mutant systems (cyan) were larger than those of the WT systems (yellow) (Figure 4D,F). In particular, the opening degree of Mps1^C604Y^/NMS-P715 proved to be significantly much larger than Mps1^C604Y^/Cpd-5. Based on the above results, the distribution of the opening degree of the A-loop for the C604W mutation, another similar point mutation, was also studied by classical MD simulations. Interestingly, the C604W mutation to these three inhibitors exhibited quite different phenomena than the RMSD values of both the proteins and inhibitors, and were more stable than those in the C604Y mutation (Figure 2B,D,F and Figure S1(A,C,E)). In addition, the distribution of the opening degree of the A-loop between Mps1^WT^ and Mps1^C604W^ bound to the inhibitors showed high similarity (Figure S1(B,D,F)). These results are consistent with a previous study which showed that Cpd-5 and NMS-P715 bind better to the C604W mutation than to the C604Y mutation, and may be due to the fact that the C604W mutation forms more stable hydrogen bonds to Trp-604 and an even closer contact to Gln-541 than the C604Y mutation [14]. However, more experimental and theoretical studies will be required to fully understand the resistance mechanism of the C604W mutation. Overall, these interesting findings indicate that the C604Y mutation induced conformational change and the flexibility of the A-loop with different degrees, and may be the main reason for the varying degrees of C604Y resistance to different inhibitors. The above findings also indicate that the C604Y mutation-induced conformational changes may be the primary driving force for the redistribution of binding free energies. When binding to the inhibitors Cpd-5/NMS-P715, the A-loop region of Mps1^C604Y^ changes into an outward-moving conformation. However, this was not observed for reversine, suggesting that the binding preference of Cpd-5/NMS-P715 to Mps1^WT^ over Mps1^C604Y^ was regulated by conformational selection or an induced-fit mechanism. Therefore, molecular mechanics/generalized Born solvent area (MM/GBSA) methodology together with enhanced samplings were performed to analyze the energetic contributions while determining the association and dissociation processes between protein and ligand.

### 2.2. Binding Profiles Revealed Through the MM/GBSA Binding Free Energy Calculations and the Residue-Ligand Interaction Spectra

To gain further information on the origination of binding free energy (Δ*G*_bind_) between protein and inhibitor, the five energy components (Δ*E*_vdW_, Δ*E*_elec_, Δ*G*_GB_, Δ*G*_SA_, TΔ*S*) were estimated on the basis of the MM/GBSA methodology. However, this could not be used to estimate the absolute experimental binding free energies in an accurate fashion, but provided high correlation with experimental values [23,24,25,26]. As listed in Table 1, Δ*G*_bind_ of Mps1^WT^/reversine, Mps1^C604Y^/reversine, Mps1^WT^/Cpd-5, Mps1^C604Y^/Cpd-5, Mps1^WT^/NMS-P715 and Mps1^C604Y^/NMS-P715 were −34.43 ± 3.02, −31.39 ± 3.66, −41.91 ± 4.22, −32.99 ± 4.34, −42.94 ± 4.40 and −25.72 ± 4.64 kcal/mol, respectively. It can be observed that the Δ*G*_bind_ shows high correlation with the reported experimental K_D_ data (Table 1), and various energy terms contribute differently to Δ*G*_bind_ for each system [14]. Herein, we only discuss the electrostatic contributions (Δ*E*_elec_) and entropic contributions (TΔ*S*). As illustrated in Table 1, the electrostatic contributions for Cpd-5/NMS-P715 between the WT systems and C604Y systems proved to be significantly different. The electrostatic contributions for Mps1^WT^/Cpd-5 and Mps1^C604Y^/Cpd-5 were −28.42 ± 3.26, −21.86 ± 6.77 kcal/mol, and those for NMS-P715 were −29.57 ± 3.35, −20.63 ± 6.7 kcal/mol. For comparison, the electrostatic contributions for reversine in Mps1^WT^ and Mps1^C604Y^ are almost identical at −26.01 ± 3.67 and −24.07 ± 3.59 kcal/mol. Furthermore, the entropic contributions for the Mps1^C604Y^ bound to Cpd-5/NMS-P715 were found to increase. The entropic contribution for Mps1^WT^/Cpd-5 and Mps1^C604Y^/Cpd-5 were −14.02 ± 4.62, −21.92 ± 5.58 kcal/mol, and those for NMS-P715 were −13.02 ± 4.57, −20.92 ± 4.34 kcal/mol. Nevertheless, the entropic contributions for reversine in Mps1^WT^ and Mps1^C604Y^ were almost identical with −10.02 ± 3.22 and −11.92 ± 4.41 kcal/mol. Overall, the decrease of the electrostatic contributions and the increase of the entropic contributions caused by the C604Y mutation may, at least in part, account for the inhibitor resistance.

**Figure 5:**
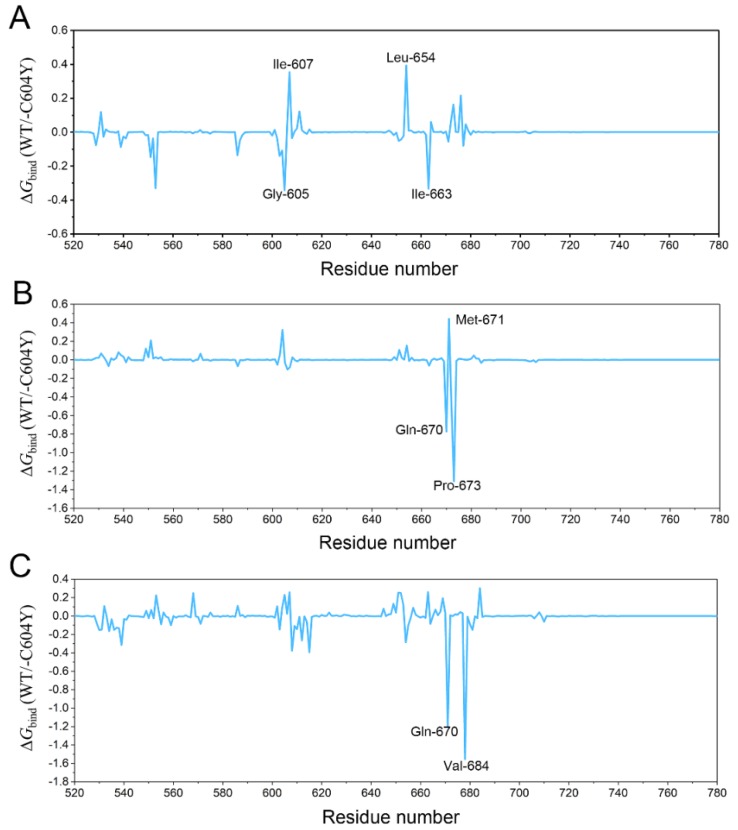
The energetic differences of the residue contributions to the binding free energies between the WT system and C604Y system (ΔΔ*G* = Δ*G*_WT_ − Δ*G*_C604Y_). (**A**) Mps1^WT^/reversine and Mps1^C604Y^/reversine; (**B**) Mps1^WT^/Cpd-5 and Mps1^C604Y^/Cpd-5; (**C**) Mps1^WT^/NMS-P715 and Mps1^C604Y^/NMS-P715.

To further explore the receptor-ligand recognition patterns and to quantitatively highlight the vital energetic distributions in inhibitor resistance, per-residue decomposition calculation was carried out using the MM/GBSA methodology. Energetic differences of per-residue between the WT system and the C604Y system (ΔΔ*G* = Δ*G*_WT_ − Δ*G*_C604Y_) are illustrated in Figure 5. The negative values indicated that the residues in the WT system formed stronger interactions with an inhibitor than those of the C604Y system. Accordingly, positive values indicated that the residues in the WT system formed weaker interactions with the inhibitor than those for the C604Y system. As illustrated in Figure 5A, energetic contributions for each residue between Mps1^WT^/reversine and Mps1^C604Y^/reversine were quite similar, with an energetic difference of less than 0.5 kcal/mol. However, the energetic contributions for Mps1^WT^/Mps1^C604Y^ bound to Cpd-5/NMS-P715 were quite different. The energetic contribution of residues in the A-loop (the residues of Gln-670, Pro-673 in Mps1^WT^/Cpd-5 and Gln-670, Val-684 in Mps1^WT^/NMS-P715) decreased when C604Y mutation occurred (Figure 5B,C). Despite the high structural similarity between Cpd-5 and NMS-P715, the C604Y mutation for NMS-P715 leads to a much larger energetic difference than that for Cpd-5 in the A-loop. It is noteworthy that the most populated conformations of inhibitors between WT and C604Y exhibit a similar pattern with minor adjustments (Figure 4B,D,F). These results are consistent with a previous study in which the inhibitors in the crystal structures between WT and C604Y mutation showed high similarity [14]. Combined with per-residue decomposition analysis, the hydrophobic and electrostatic interactions between the A-loop and inhibitors may contribute to altered affinity. Overall, the resistance of inhibitors may be related to the large conformational change of the A-loop induced energetic redistributions.

### 2.3. Accelerated MD (aMD) Simulations

**Figure 6:**
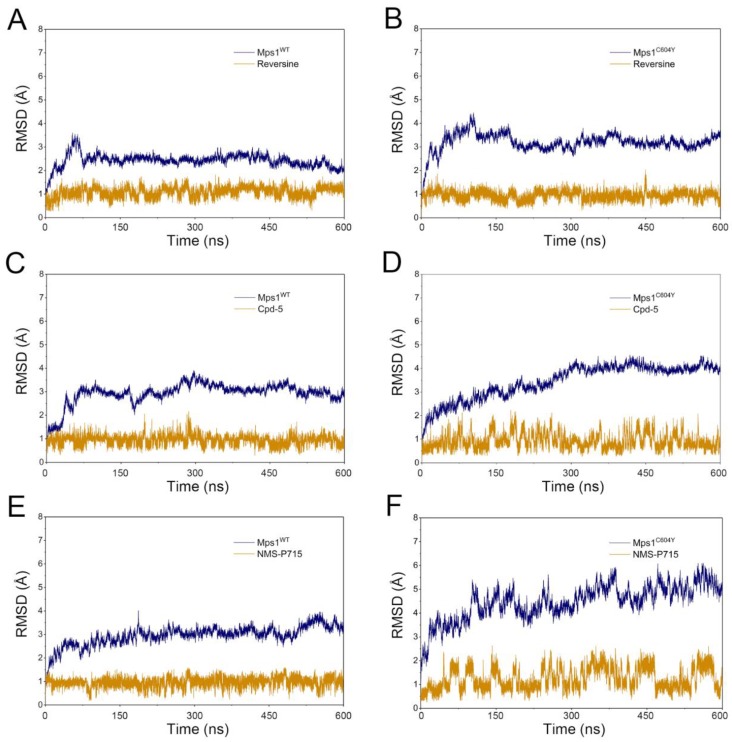
RMSD analysis of reversine, Cpd-5 and NMS-P715 in Mps1^WT^ and Mps1^C604Y^ from accelerated molecular dynamic (aMD) simulations. (**A**) Mps1^WT^/reversine; (**B**) Mps1^C604Y^/reversine; (**C**) Mps1^WT^/Cpd-5; (**D**) Mps1^C604Y^/Cpd-5; (**E**) Mps1^WT^/NMS-P715; (**F**) Mps1^C604Y^/NMS-P715.

**Figure 7:**
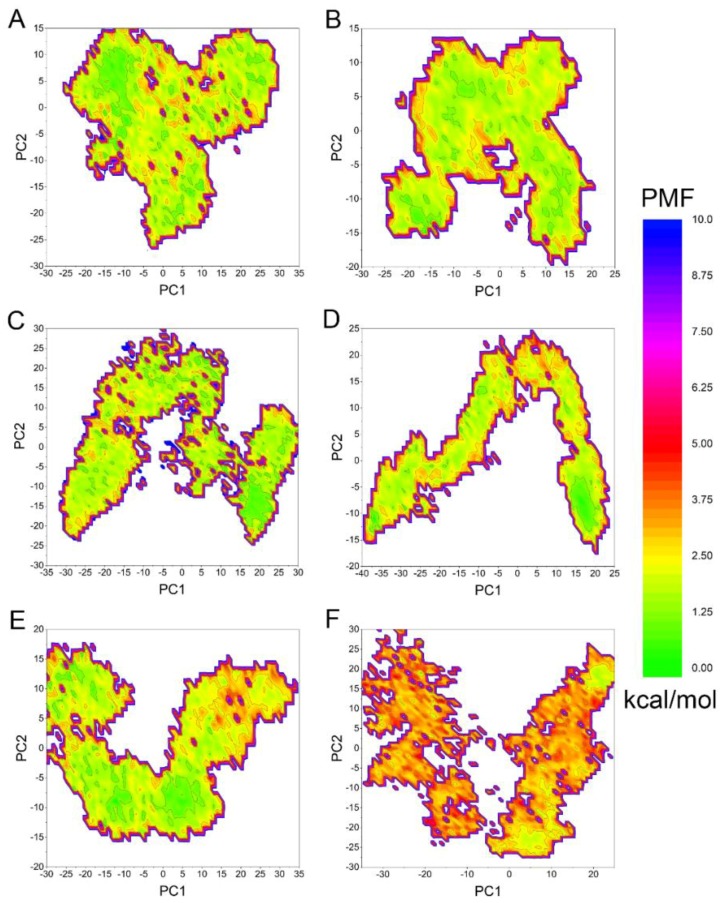
Free energy map calculated from the projection of the structures extracted from aMD simulations on the main components PC1 and PC2. (**A**) Mps1^WT^/reversine; (**B**) Mps1^C604Y^/reversine; (**C**) Mps1^WT^/Cpd-5; (**D**) Mps1^C604Y^/Cpd-5; (**E**) Mps1^WT^/NMS-P715; (**F**) Mps1^C604Y^/NMS-P715.

Accelerated molecular dynamic simulation has been successfully used for numerous of systems and hundreds of nanosecond aMD simulations have been demonstrated to exhibit millisecond-timescale events [27,28]. To speed up the sampling and in order to further confirm the stability of conformations calculated in classical MD simulations, aMD simulations on Mps1^WT^/reversine, Mps1^C604Y^/reversine, Mps1^WT^/Cpd-5, Mps1^C604Y^/Cpd-5, Mps1^WT^/NMS-P715, and Mps1^C604Y^/NMS-P715 were carried out. As illustrated in Figure 6, the RMSD evolution of the backbone atoms of proteins for all systems tended to converge after ~100–300 ns in the aMD simulations. This finding suggests that all of the simulated systems became stable enough through 600 ns of aMD simulations. During the aMD simulations, the RMSDs of all inhibitors in the WT systems were relatively stable (Figure 6A,C,E); however, the RMSDs of Cpd-5/NMS-P715 in Mps1^C604Y^ were more fluctuant in comparison with reversine (Figure 6B,D,F). These findings suggested that C604Y mutation may significantly destabilize the Cpd-5/NMS-P715 bound to the binding pocket. Thereafter, the trajectories from aMD simulations in conjunction with principal component analysis (PCA) were performed to recover original free energy maps (Figure 7). As shown in Figure 7A,C,E, there were only one or two energetic deep wells for both the inhibitors bound to Mps1^WT^, and the energetic deep well for reversine bound to Mps1^C604Y^ was also mainly concentrated in one region (Figure 7B). However, the energetic wells for Cpd-5/NMS-P715 in Mps1^C604Y^ were concentrated in numerous regions and it was difficult to sample the lowest energy minimum as well as the WT systems (Figure 7D,F). These energetic wells corresponded to the unstable conformations of Mps1^C604Y^ bound to Cpd-5/NMS-P715 during aMD simulations. Furthermore, Mps1^C604Y^/NMS-P715 exhibited a higher potential of mean force (PMF) than Mps1^C604Y^/Cpd-5. NMS-P715 proved to be more difficult than Cpd-5 in sampling of the lowest energy minimum when C604Y mutation occurred. The above results are in good agreement with the results obtained from classical MD simulation. This indicates that the C604Y mutation affects the stability between protein and inhibitor, resulting in a different degree of resistance to inhibitors.

### 2.4. Dissociation Processes Revealed by US Simulations

Unfortunately, classical MD simulations are not useful to characterize a physically associated pathway on how a ligand binds to or dissociates from its receptor. To gain further information on the dissociation processes of reversine, Cpd-5, NMS-P715 from Mps1^WT^ and Mps1^C604Y^, US simulations were used to describe the dissociation pathway of an inhibitor towards its receptor. The US simulation represents one of the most classically enhanced sampling methodologies able to cancel the conformational sampling trap into local minima by addition of biasing potentials on the reaction coordinate (RC) [29].

**Figure 8:**
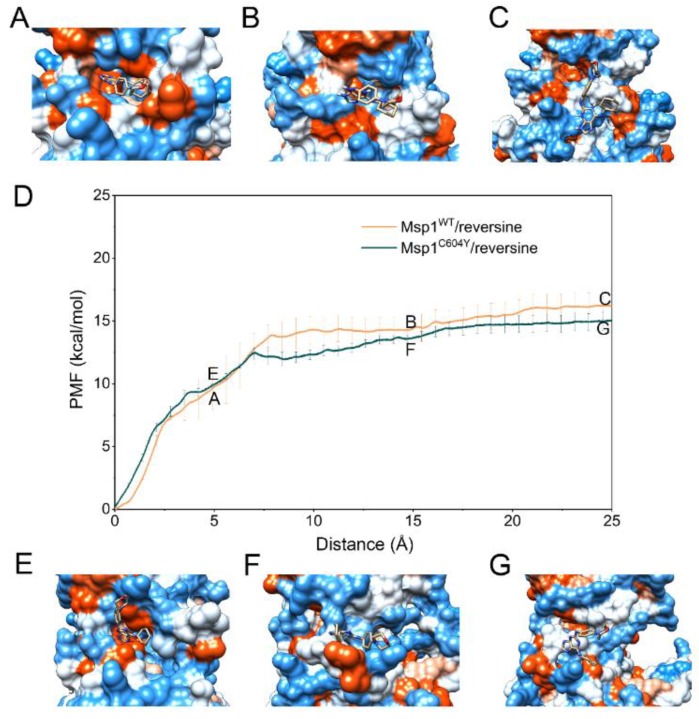
Alignment of the PMFs for reversine dissociate from Mps1^WT^ and Mps1^C604Y^.

**Figure 9:**
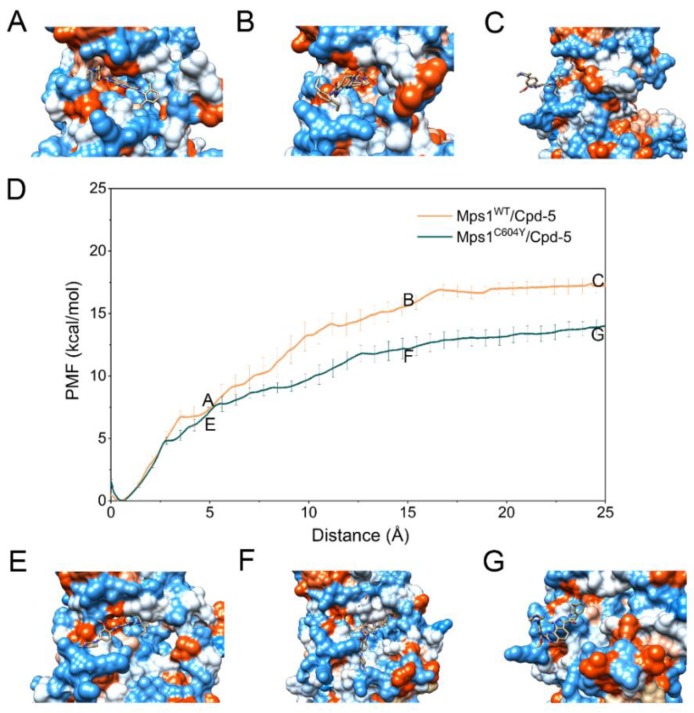
Alignment of the PMFs for Cpd-5 dissociate from Mps1^WT^ and Mps1^C604Y^.

**Figure 10:**
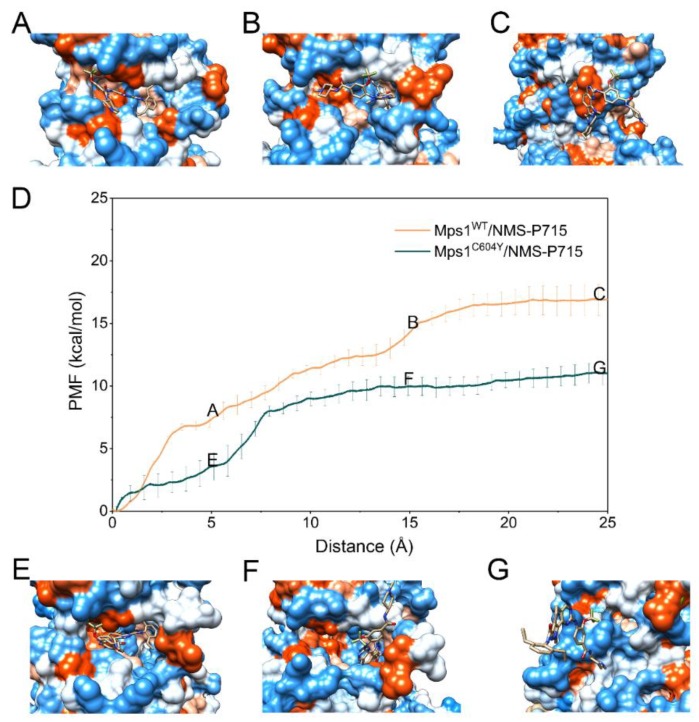
Alignment of the potential of mean forces (PMFs) for NMS-P715 dissociate from Mps1^WT^ and Mps1^C604Y^.

The PMF depth (Δ*W*_PMF_), used synonymously with binding free energy profiles in the literature, evaluates how binding free energy values change as a function of given RCs. Δ*W*_PMF_ for Mps1^WT^/reversine, Mps1^C604Y^/reversine, Mps1^WT^/Cpd-5, Mps1^C604Y^/Cpd-5, Mps1^WT^/NMS-P715 and Mps1^C604Y^/NMS-P715 were −16.05 ± 0.16, 14.88 ± 0.09, −17.14 ± 0.09, −13.97 ± 0.43, −16.81 ± 0.08, and −10.89 ± 0.17 kcal/mol, respectively (Table 1). The Δ*W*_PMF_ was found to be in good agreement with the K_D_ value from experimental data and correctly ranked (Table 1). Therefore, the PMF curves were used to provide further information on the different dissociation processes of reversine, Cpd-5, NMS-P715 towards Mps1^WT^ and Mps1^C604Y^ from the binding pocket. The PMF profiles of reversine, Cpd-5, NMS-P715 dissociating from the binding sites of the Mps1^WT^/Mps1^C604Y^ systems were quite different (Figure 8, Figure 9 and Figure 10). We could observe that the PMF curves for reversine in Mps1^WT^ exhibited no significant difference from that in Mps1^C604Y^ if the calculation error was considered (Figure 8D), but those for Cpd-5, NMS-P715 towards Mps1^WT^ and Mps1^C604Y^ exhibited quite different behaviors (9D and 10D). As shown in Figure 8D, the PMF curve of Mps1^WT^/reversine was similar to that for Mps1^C604Y^/reversine, indicating a similar energy potential depth, and thus similar residence time. During this process, the binding poses of reversine in the WT system and the C604Y mutant system were quite similar (Figure 8A–C,E–G). For the inhibitors Cpd-5/NMS-P715, the PMF curve of both in the WT systems and C604Y mutant systems increased markedly at first (RC was 0–16 Å). Presumably, here (RC was 16 Å) the inhibitor moved to the entrance of the binding site, with the substructure adjusting accordingly (9A–G and 10A–G). Distinct from that of reversine, the PMF curves for the WT systems of Cpd-5/NMS-P715 were relatively higher than those of the corresponding C604Y mutation systems (9D and 10D). In addition, the differences of Δ*W*_PMF_ values for NMS-P715 between WT and C604Y mutation were much larger than those for Cpd-5 (9D and 10D). Previous studies proposed that the higher the PMF curve, the more free energy was released during the ligand dissociate from the target, thus longer residence time [30]. In other words, the C604Y mutation could induce resistance to both Cpd-5 and NMS-P715, but exhibited only slight impact on reversine. This latter notion was consistent with the experimental data that Cpd-5 and NMS-P715 bound to C604Y mutation with a significantly worse K_D_ value, but retains affinity for reversine (Table 1). In summary, the US simulations provided details of the dissociation processes and were feasible to develop novel inhibitors to improve the ability to combat resistance.

## 3. Conclusions

To investigate the resistance mechanism of the Mps1 C604Y mutation, a computational strategy combining all-atom classical MD simulation, MM/GBSA binding free energy calculations and energy decomposition, aMD simulations, and US simulations was carried out to shed light on the physical and molecular principles via the three well-studied inhibitors reversine, Cpd-5, and NMS-P715 with different inhibitions towards WT and C604Y mutation systems. The classical MD simulation results unambiguously revealed that the binding preferences of inhibitors Cpd-5/ NMS-P715 to Mps1^WT^ over Mps1^C604Y^ were regulated by the conformational change of the A-loop and the variances were due to the electrostatic interactions and conformational entropy. Accelerated MD simulations provided a highly effective way to search possible conformations of inhibitors in the binding pockets of both Mps1^WT^ and Mps1^C604Y^. The results of aMD simulations indicated that the C604Y mutation influenced the stability between the protein and the inhibitor, resulting in a different degree of resistance to inhibitors. Additionally, US simulations were used as a proof of principle of the dissociation processes of inhibitors from Mps1^WT^ and Mps1^C604Y^. Δ*W*_PMF_ estimated from the US simulations were in good agreement with the experimental K_D_ data (Table 1) and revealed a shorter residence time may be responsible for inhibitor resistance. Overall, the obtained resistance mechanisms may guide rational screening and help in the design of novel Mps1 inhibitor to combat inhibitor resistance.

## 4. Materials and Methods

### 4.1. Initial Structures of Mps1^WT^/reversine, Mps1^WT^/Cpd-5, and Mps1^WT^/NMS-P715

The atomic coordinates of the Mps1^C604Y^/reversine (2-(4-morpholinoanilino)-6-cyclohexylaminopurine), Mps1^C604Y^/Cpd-5 (*N*-(2,6-diethylphenyl)-8-((2-methoxy-4-(4-methylpiperazin-1-yl)phenyl)amino)-1-methyl-4,5,5a,9a-tetrahydro-1H-pyrazolo[4,3-h]quinazoline-3-carboxamide), Mps1^C604Y^/NMS-P715 (*N*-(2,6-diethylphenyl)-1-methyl-8-((4-((1-methylpiperidin-4-yl)carbamoyl)-2-(trifluoromethoxy)phenyl)amino)-4,5,5a,9a-tetrahydro-1H-pyrazolo[4,3-h]quina- zoline-3-carboxamide), Mps1^C604W^/Cpd-5 were downloaded from Protein Data Bank (PDB) database (PDB code: 5LJJ, 5NTT, 5MRB, 5O91) [14,31]. The Mps1^WT^/NMS-P715 (PDB code: 2X9E) complex was modeled rather than downloaded from the PDB database due to the A-loop in the crystal structure being unresolved, and the A-loop playing a vital in signal transduction. Therefore, the structures of Mps1^WT^/reversine, Mps1^WT^/Cpd-5 and Mps1^WT^/NMS-P715, Mps1^C604W^/reversine, Mps1^C604W^/NMS-P715 were mutated by Chimera software (version 1.12) (University of California, San Francisco, CA, USA). Thereafter, the structures were refined, including modelling the A-loop and missing side-chains by the “model/refine loop” module in Chimera software (version 1.12). Then, all non-bonded hetero-atoms and water molecules were removed, adding the missing hydrogen atoms via Chimera software (version 1.12) [32].

### 4.2. Classical MD Simulations

The crystal structures of Mps1^C604Y^/reversine, Mps1^C604Y^/Cpd-5, Mps1^C604Y^/NMS-P715, Mps1^C604W^/Cpd-5 and modeled structures of Mps1^WT^/reversine, Mps1^WT^/Cpd-5 and Mps1^WT^/NMS-P715, Mps1^C604W^/reversine, Mps1^C604W^/NMS-P715 were selected as the initial structures for the classical MD simulations. Reversine, Cpd-5, and NMS-P715 were optimized by the Hartree–Fock (HF) method based on 6–31G* basis set (Gaussian, Inc., Wallingford, CT, USA). Then, the partial atomic charges for these inhibitors were fitted by the restrained electrostatic potential (RESP) fitting methods implemented in an antechamber module in the Assisted Model Building with Energy Refinement (Amber) 16 simulation package. The force field parameters of the proteins and inhibitors were described by the Amber ff14SB force field and General Amber Force Field 2 (GAFF2), respectively [33,34]. After that, each system was solvated using a TIP3P water box with a 15 Å distance between the complex surface and the box boundary. Finally, an appropriate number of counter ions were added to neutralize each system.

Prior to the productive classical MD simulations, the molecular mechanics (MM) minimization, heating and equilibration were carried out by the Sander module in the Amber 16 package. Initially, a three-step energy minimization procedure was performed. Initially, all the water molecules were restrained by a harmonic constraint potential with 10 kcal mol^−1^ Å^−2^. After that, the side chains of proteins were minimized in order to adjust structural clash by constraining the backbone atoms of the protein with 10 kcal mol^−1^ Å^−2^ of harmonic constraint potential. Lastly, all atoms in the solvated box were minimized with no restraint. During each MM minimization phase, 5000 steps of steepest descent method and 5000 steps of conjugated gradient method were applied. Subsequently, each system was gradually heated up from 0 to 300 K for 200 ps with a harmonic constraint potential of 10.0 kcal mol^−1^ Å^−2^ in the canonical (NVT) ensemble. After heating, 1 ns equilibration were performed to optimize the solvent density in the NPT ensemble (Temperature = 300 K and Pressure = 1 atm) by restraining all heavy atoms. Ultimately, all systems were submitted to 200 ns classical MD simulation in the NPT ensemble (Temperature = 300 K and Pressure = 1 atm) without any restraint. During the productive classical MD simulations, Particle Mesh Ewald (PME) algorithm was applied to compute the long-range electrostatic interactions [35]. The SHAKE algorithm was utilized to equilibrium the length of hydrogen atoms involved in the covalent bonds and the distance of non-bonded cutoff was set as 10.0 Å to treat non-bonding interactions [36]. The temperature of each simulated system was maintained by the Langevin temperature equilibration scheme and the pressure was maintained by the Berendsen barostat [37,38,39]. The coordinates were recorded every 2 ps for all the production trajectories.

### 4.3. DCC Analysis

The DCC map (*C_ij_*) between residues *i* and *j* were computed based on a total of 10,000 snapshots from last 40 ns classical MD simulation trajectories by using the CPPTRAJ module in Amber 16 package [40]. The DCC map (*C_ij_*) was calculated by the following equation:

(1) $$\;C_{ij}=\frac{\langle r_{i}r_{j}\rangle }{\sqrt{\langle r_{i}{}^{2}r_{j}{}^{2}\rangle }}$$

where *C_ij_* represents the cross-correlation matrix of C_α_ atom between residues *i* and *j* that relative to their average positions, Δ*r_i_* or Δ*r_j_* is the displacement from the mean position of the *i*th or *j*th atom. 〈〉 denotes the time average.

### 4.4. Binding Free Energy Calculations

The extensively validated MM/GBSA methodology can provide relative quantitative predictions of binding free energies between protein and ligand as the following equations [39,40,41,42]:

(2) $$\Delta G_{bind}\;=\;\Delta G_{\mathrm{Rec}+\mathrm{Lig}}\;-\;\left(\Delta G_{\mathrm{Rec}}+\;\Delta G_{\mathrm{Lig}}\right)\;=\;\Delta E_{\mathrm{MM}}\;+\;\Delta G_{\mathrm{sol}}\;-\;T\Delta S$$

(3) $$\Delta E_{\mathrm{MM}}=\Delta E_{\mathrm{int}}+\Delta E_{\mathrm{vdW}}+\Delta E_{\mathrm{elec}}$$

(4) $$\Delta G_{\mathrm{sol}}=\Delta G_{\mathrm{GB}}+\Delta G_{\mathrm{SA}}$$

where Δ*G*_Rec+Lig_, Δ*G*_Rec_, and Δ*G*_Lig_ represent the binding free energies of receptor-ligand complex, receptor and ligand, respectively (Equation (2)). Δ*E*_MM_ and Δ*G*_sol_ represent the molecular mechanics (MM) interaction and solvation energy. TΔ*S* is the conformational entropy (translational, rotational, and vibrational terms) at temperature T. The Δ*E*_MM_ is given as the sum of three terms (Equation (3)): inter-molecular interaction energy (Δ*E*_int_), van der Waals energy (Δ*E*_vdW_), electrostatic energy (Δ*E*_elec_). In Equation (4), the solvation free energy consists of polar (Δ*G*_GB_) and nonpolar contributions (Δ*G*_SA_). In this study, the Δ*G*_SA_ was calculated by using the solvent accessible surface area (SASA) model via the LCPO algorithm. The Δ*G*_GB_ was estimated by using a Generalized-Boltzmann (GB) model proposed by Onufriev et al. (igb = 2) [43]. The TS was estimated by normal mode analysis (NMA) [44]. In this study, Δ*G*_bind_ were computed by using the classical MD simulation trajectories between 160 ns and 200 ns with 500 snapshots.

### 4.5. Accelerated MD (aMD) Simulations

The last snapshots from classical MD simulations were selected as the initial structures for the aMD simulations. All the systems were submitted to 10 ns equilibration within the NPT collective, and then, 20 ns equilibration in the NVT collective. Ultimately, 600 ns aMD simulations were carried out by the dual-boost approach to enhance the sampling, including a total boost potential and a dihedral boost potential. The boost potential *ΔV(r)* modifies the original potential energy surface is defined in equations as follows [45]:

(5) $$\begin{array}{ccc} \Delta V\left(r\right)=0 & \; & \Delta V\left(r\right)\geq E \end{array}$$

(6) $$\begin{array}{ccc} \Delta V\left(r\right)=\frac{{\left[E-\Delta V\left(r\right)\right]}^{2}}{\alpha +\left[E-\Delta V\left(r\right)\right]} & \; & \Delta V\left(r\right)<E \end{array}$$

where *α* and *E* represent the acceleration factor and the threshold energy, respectively. The boost potential was on the basis of the total number of atoms, dihedrals, and average energies computed from the first 10 ns of the classical MD simulations. During the aMD simulations, PME algorithm with a cutoff of 10.0 Å was used to deal with the long-range electrostatic interactions. The SHAKE algorithm were utilized to equilibrium the length of hydrogen atoms involved in the covalent bonds. The temperature of each simulated system was maintained by the Langevin temperature equilibration scheme [35,36,39]. The aMD simulation coordinates were recorded every 1 ps for subsequent analysis. After aMD simulations, the cumulant expansion to the second order was utilized to calculate free energy map. The modified potential energy in conjunction with the principal component 1 (PC1) and principal component 2 (PC2) calculated by PCA were applied to recover the original free energy map. PCA is generally applied to dimensionality reduction of the data and recognize diverse conformations the protein attains during MD simulation. In this study, the 3N × 3N covariance matrix was created by superposition of the structures from aMD simulations, including remove translational and rotational motions of all C_α_ atoms. The sets of eigenvectors and eigenvalues were generated by the diagonalization of the covariance matrix by CPPTRAJ module in Amber 16 package [40,46].

### 4.6. US Simulations

The structures of last snapshot obtained from the classical MD simulations were selected as the initial structures for the subsequent US simulations. The possible directions of the reaction coordinates (RCs) along the ATP channel were recognize via the CAVER Analyst 1.0 software (Masaryk University, Brno, Czech Republic) and the largest binding pocket direction was selected as the unbinding direction for US simulation [47]. The RC for each system was extended 25 Å from the initial position and divided into 51 continuous windows with 0.5 Å/window. For each system, 10 ns US simulations were carried out for each window to ensure the convergence. In addition, the harmonic potential was applied to each window. An elastic constant of 10 kcal mol^−1^ Å^−2^ was employed to each window to pull each inhibitor away from the binding site of Mps1 at constant speed and force. Finally, the data were collected from separate simulation windows and the PMF profile were computed by the weighted histogram analysis method (WHAM) software (University of Pittsburgh, Pittsburgh, PA, USA) by three replica simulations [48].

## Figures and Tables

**Table 1:** Energy terms of reversine, Cpd-5 and NMS-P715 towards Mps1^WT^ and Mps1^C604Y^ (kcal/mol).

Name	Reversine		Cpd-5		NMS-P715	
	Mps1^WT^	Mps1^C604Y^	Mps1^WT^	Mps1^C604Y^	Mps1^WT^	Mps1^C604Y^
Δ*E*_vdW_	54.07 ± 2.93	51.87 ± 3.47	68.01 ± 4.41	−66.45 ± 3.7	70.88 ± 3.18	−71.32 ± 4.8
Δ*E*_elec_	26.01 ± 3.67	24.07 ± 3.59	28.42 ± 3.26	21.86 ± 6.77	29.57 ± 3.35	−20.63 ± 6.7
Δ*G*_GB_	41.86 ± 3.05	39.58 ± 2.91	48.95 ± 3.63	51.61 ± 2.39	52.95 ± 7.66	54.11±5.94
Δ*G*_SA_	−6.22 ± 0.27	−6.02 ± 0.31	−8.37 ± 0.39	−8.21 ± 0.33	−8.45 ± 0.40	−8.78 ± 0.50
TΔ*S*	10.02 ± 3.22	11.92 ± 4.41	14.02 ± 4.62	21.92 ± 5.58	13.02 ± 4.57	20.92 ± 4.34
Δ*G*_bind_	34.43 ± 3.02	31.39 ± 3.66	41.91 ± 4.22	32.99 ± 4.34	42.94 ± 4.40	25.72 ± 4.64
Δ*W*_PMF_	16.05 ± 0.16	14.88 ± 0.09	17.14 ± 0.09	13.97 ± 0.43	16.81 ± 0.08	10.89 ± 0.17
K_D_ (nM)	41 ± 21	86 ± 24	1.6 ± 0.2	471 ± 50	4.7 ± 2.5	1764 ± 204

Δ*E*_vdW_: Van der Waals energy; Δ*E*_elec_: Electrostatic energy; Δ*G*_GB_: Polar contribution to solvation; Δ*G*_SA_: Non-polar contribution to solvation; Δ*G*_bind_: Binding free energy; Δ*W*_PMF_: PMF depth based on 20–25 Å along the reaction coordinate; K_D_: Dissociation constant.

## Supplementary Materials

The following are available online at http://www.mdpi.com/1420-3049/23/6/1488/s1, Figure S1: RMSD analysis of Mps1^C604W^/reversine, Mps1^C604W^/Cpd-5 and Mps1^C604W^/NMS-P715 from classical MD simulations. The distribution of the opening degree of the A-loop between Mps1^WT^/reversine, Mps1^C604W^/reversine, Mps1^WT^/Cpd-5 and Mps1^C604W^/Cpd-5 and Mps1^WT^/NMS-P715 and Mps1^C604W^/NMS-P715.
