# Supplementary material for: Insights into Resistance Mechanisms of Inhibitors to Mps1 C604Y Mutation via a Comprehensive Molecular Modeling Study
Source: Molecules. 2018 Jun 20;23(6):1488. doi: 10.3390/molecules23061488 (PMC6100145; doi:10.3390/molecules23061488)
Supplement: Supplementary file 1 [file molecules-23-01488-s001.pdf]

SUPPLEMENTARY MATERIAL

Insights into Resistance Mechanisms of Inhibitors to Mps1 C604Y Mutation via a Comprehensive Molecular Modeling Study

Yuan Chen [1,2,†], Wenquan Yu [2,†], Cui-cui Jiang [2] and Jin-gui Zheng [1,*]

[1] College of Crop Science, Fujian Agriculture and Forestry University, Fuzhou 350003, China; katecy@163.com
[2] Institute of Agricultural Engineering and Technology, Fujian Academy of Agricultural Sciences, Fuzhou 350003, China; ywq333@163.net (W.Y.); yumin793@163.com (C.-C.J.)
* Correspondence: jingui_zheng@yeah.net; Tel./Fax: 86-0591-8343-8961
† These authors contributed equally to this work

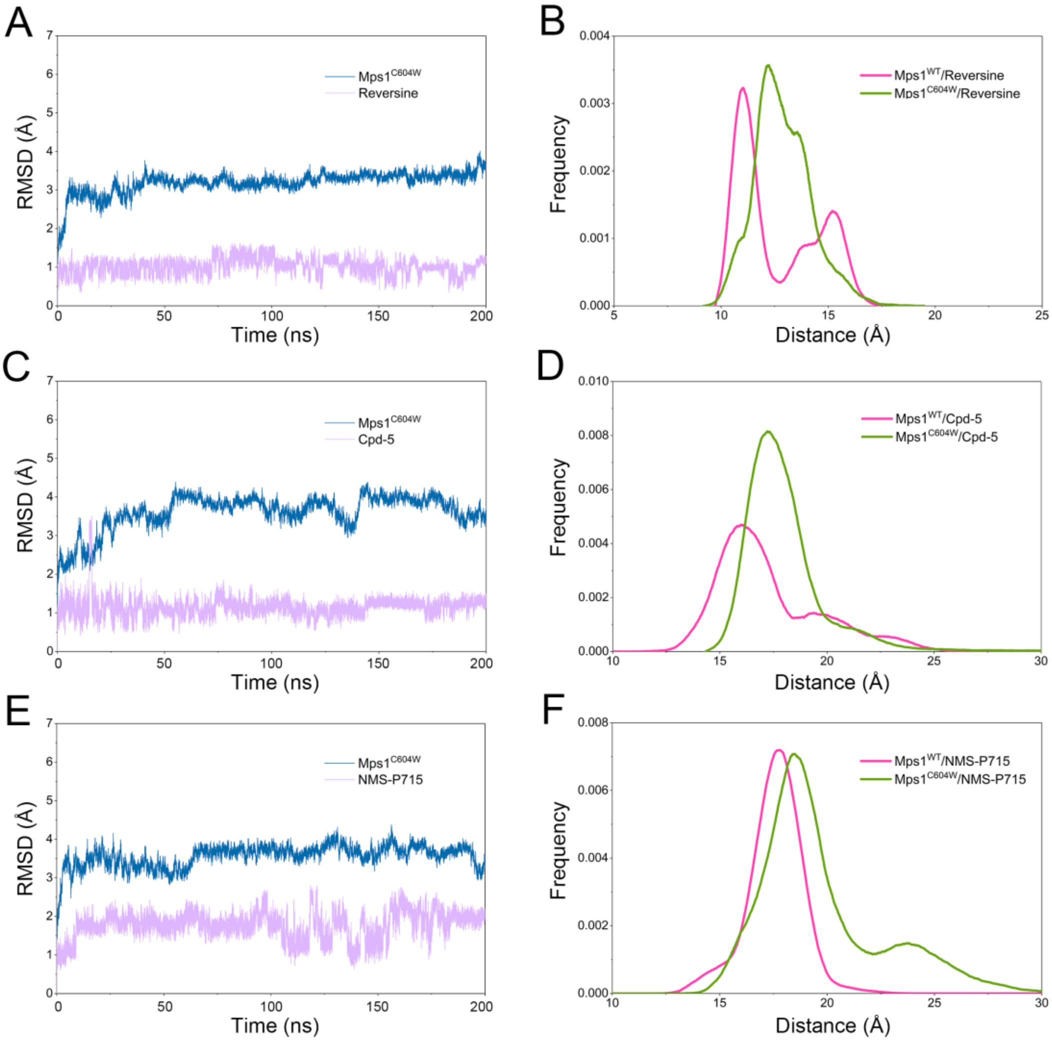

Figure S1. RMSD analysis of (**A**) Mps1^{C604W}/reversine, (**C**) Mps1^{C604W}/Cpd-5, (**E**) Mps1^{C604W}/NMS-P715 from classical MD simulations. The distribution of the opening degree of the A-loop between (**B**) Mps1^{WT}/reversine and Mps1^{C604W}/reversine, (**D**) Mps1^{WT}/Cpd-5 and Mps1^{C604W}/Cpd-5, (**F**) Mps1^{WT}/NMS-P715 and Mps1^{C604W}/NMS-P715.
